# Fabrication of a Microfluidic-Based Device Coated with Polyelectrolyte-Capped Titanium Dioxide to Couple High-Performance Liquid Chromatography with Inductively Coupled Plasma Mass Spectrometry for Mercury Speciation

**DOI:** 10.3390/polym16162366

**Published:** 2024-08-21

**Authors:** Ji-Hao Chen, Yu-Ting Luo, Yi-An Su, Yan-Ren Ke, Ming-Jay Deng, Wei-Yu Chen, Cheng-Yu Wang, Jia-Lin Tsai, Cheng-Hsing Lin, Tsung-Ting Shih

**Affiliations:** 1 Department of Chemistry, Fu Jen Catholic University, New Taipei City 242062, Taiwan; wsx852dd@gmail.com (J.-H.C.); allenke0511@gmail.com (Y.-R.K.); s930170280227@yahoo.com (C.-Y.W.); jialintsai2002@gmail.com (J.-L.T.); 2 Department of Biomedical Engineering and Environmental Sciences, National Tsing Hua University, Hsinchu 300044, Taiwan; ytluo693@gmail.com (Y.-T.L.); lynn79827@gmail.com (Y.-A.S.); maxlin.lf@gmail.com (C.-H.L.); 3 Department of Applied Chemistry, Providence University, Taichung City 433303, Taiwan; dengmj1020@pu.edu.tw; 4 Department of Materials Engineering, National Pingtung University of Science and Technology, Pingtung County 912301, Taiwan; wychen@mail.npust.edu.tw

**Keywords:** mercury (Hg) speciation, microfluidic-based device, poly(methyl methacrylate) (PMMA), titanium dioxide nanoparticles (nano-TiO_2_s), poly(diallyldimethylammonium chloride) (PDADMAC), inductively coupled plasma mass spectrometry (ICP-MS)

## Abstract

Mercury (Hg) is a toxic element which impacts on biological systems and ecosystems. Because the toxicity of Hg species is highly dependent on their concentration levels and chemical forms, the sensitive identification of the chemical forms of Hg—i.e., Hg speciation—is of major significance in providing meaningful information about the sources of Hg exposure. In this study, a microfluidic-based device made of high-clarity poly(methyl methacrylate) (PMMA) was fabricated. Then, titanium dioxide nanoparticles (nano-TiO_2_s) were attached to the treated channel’s interior with the aid of poly(diallyldimethylammonium chloride) (PDADMAC). After coupling the nano-TiO_2_-coated microfluidic-based photocatalyst-assisted reduction device (the nano-TiO_2_-coated microfluidic-based PCARD) with high-performance liquid chromatography (HPLC) and inductively coupled plasma mass spectrometry (ICP-MS), a selective and sensitive, hyphenated system for Hg speciation was established. Validation procedures demonstrated that the method could be satisfactorily applied to the determination of mercury ions (Hg^2+^) and methylmercury ions (CH_3_Hg^+^) in both human urine and water samples. Remarkably, the zeta potential measured clearly indicated that the PDADMAC-capped nano-TiO_2_s with a predominance of positive charges indeed provided a steady force for firm attachment to the negatively charged device channel. The cause of the durability of the nano-TiO_2_-coated microfluidic-based PCARD was clarified thus.

## 1. Introduction

Mercury (Hg) is an element naturally found in the earth’s crust and is highly toxic to living organisms and the environment [1]. Due to the enormous environmental and biological impacts caused by Hg exposure, monitoring of Hg levels in both biological systems or ecosystems has long been recognized as a critical issue all over the world. Over the past few decades, competent authorities in several countries have developed a comprehensive body of legislation governing the maximum permissible levels of Hg [2,3,4]. However, Hg can be found in many forms, including elemental mercury (Hg^0^), the mercury ion (Hg^2+^), the methylmercury ion (CH_3_Hg^+^), and so on, both in the environment and in living organisms. The toxicity of Hg species is highly dependent on their concentration levels and chemical forms [5,6]. For example, Hg in its inorganic form binding with a methyl group is known to be a developmental neurotoxin, i.e., CH_3_Hg^+^, with efficient absorption by the gastrointestinal tract and easy transport across cellular membranes due to its intensive lipophilicity [7]. In addition, the determination of Hg species is typically challenging due to the low levels of each species in most instances [8]. Therefore, the sensitive identification of the chemical forms for Hg, i.e., Hg speciation, is of major significance in providing meaningful information about the sources of Hg exposure.

Nowadays, inductively coupled plasma mass spectrometry (ICP-MS) with superior analytical features (e.g., low detection limits, wide linear dynamic range, ultrahigh sensitivity, and so on) is widely recognized as one of the most powerful methods for element determination [9,10]. Even so, the same elements in different chemical forms cannot be simultaneously identified by ICP-MS instrumentation due to the completed dissociation of sample analytes achieving during ICP atomization. Although combinations of separation techniques such as high-performance liquid chromatography (HPLC) with ICP-MS detection are frequently utilized for Hg speciation [11,12,13,14], there is a growing awareness of the impropriety of the direct introduction of salt- or organic matter-rich effluent from HPLC into ICP-MS instrumentation [15,16,17].

Up to the present, several vapor generation (VG) techniques have been proposed to interface with HPLC and ICP-MS for efficient sample introduction. The volatile products of the target analytes obtained by VG techniques are separated from liquid sample matrices for subsequent measurement, leading to the advantages of the alleviation of matrix effects and enhancement of analyte transportation [18,19]. Among the VG techniques available, photoinduced VG techniques have emerged as alternatives to those using chemical reducing agents (e.g., sodium tetrahydroborate (NaBH_4_) or tin chloride (SnCl_2_)) [20]. In 2003, Sturgeon’s group initiated related research into the development of photoinduced VG for elemental analysis [21]. Target analytes can be transformed into gaseous species with the aid of free radicals generated by the dissociation of low molecular weight (M_w_) organic acids (e.g., formic acid (HCOOH), acetic acid (CH_3_COOH), and so on) under “pure” ultraviolet (UV) irradiation. The determination of Hg based on such an analytical strategy was then successfully achieved by Hou’s group [22,23,24,25]. In 2004, Wang et al. further proposed a new methodology combining the photocatalysts titanium dioxide nanoparticles (nano-TiO_2_s) with “pure” photoinduced VG, i.e., the nano-TiO_2_-enhanced photoinduced VG technique, for analytical sensitivity improvement [26]. Afterward, different photocatalysts such as bare/silver-modified nano-TiO_2_s and zirconium dioxide nanoparticles were applied in similar studies for the same purpose [27,28]. Furthermore, due to illumination being the key to the initiation of photocatalytic reactions, quartz with excellent optical properties is often used in the fabrication of photoreactors. In other words, the expensive nature of quartz reactors continues to limit the widespread use of the two photoinduced VG techniques.

In the following years, Sun’s group made a lot of effort to explore the possibilities of using various materials (e.g., poly(tetrafluoroethylene) (PTFE) [29,30,31,32] and Pyrex glass [33]) in reactor fabrication for the development of the nano-TiO_2_-enhanced photoinduced VG technique. However, because the wavelength of the optimum transmittance of reactors did not match that of the photocatalytic activation reaction, the analytical sensitivity was thus limited. Such compromised results were unsatisfactory, even if the fabrication cost of the photoreactors was dramatically reduced. Additionally, both tangle-prone reactors and fragile tubes are troublesome issues during operation procedures.

The deadlock regarding the photoinduced VG technique has subsequently been broken by the emergence of suitable microfluidic system designs. In 2013, Shih et al. first developed a poly(methyl methacrylate) (PMMA) reactor, i.e., a microfluidic-based, photocatalyst-assisted reduction device (microfluidic-based PCARD) with outstanding clarity [34]. Meanwhile, additional accessories were eliminated because operational functionalities like mixing tees were integrated into a microfluidic system. After coupling HPLC separation with ICP-MS detection, a selective and sensitive speciation technique for the two inorganic selenium (Se) species was constructed. In view of the overconsumption of photocatalysts resulting from consecutive loading during analytical procedures, a microfluidic-based PCARD coated with nano-TiO_2_ catalysts was developed thus [35]. To simplify the coating procedure and improve the stability of the coating materials, a charge-rich polyelectrolyte, namely poly(diallyldimethylammonium chloride) (PDADMAC), was employed to cap nano-TiO_2_ catalysts for firm attachment to the treated channel interior. In other words, a PDADMAC-capped nano-TiO_2_ catalyst can be tightly embedded on the selected substrate via strong electrostatic attraction for the development of efficient VG. Although the applicability of the nano-TiO_2_-coated microfluidic-based PCARD has been demonstrated [35], the working theory of the coating method has not yet been clarified. Therefore, this work aimed to verify the properties of the PDADMAC-capped nano-TiO_2_ catalyst and further apply the established system to Hg speciation with human urine and environmental water samples.

## 2. Materials and Methods

### 2.1. Chemicals and Materials

All chemicals were of analytical reagent grade and used as received without further treatment unless otherwise stated. High-purity water was obtained by using a Milli-Q system (Millipore, Bedford, MA, USA). Hydrochloric acid (HCl; 36.5–38.0%), methanol (CH_3_OH, ≥99.9%), nitric acid (HNO_3_, 69.0–70.0%), sodium dodecyl sulfate, and sodium hydroxide (NaOH) were obtained from J.T. Baker (Phillipsburg, NJ, USA). Acetic acid (≥99.7%), ammonium acetate (≥97%), ammonium hydroxide (NH_4_OH, 30–33%), L-cysteine (≥98.5%), formic acid (HCOOH, ≥98%), 2-mercaptoethanol (≥99.0%), methylmercury(II) chloride (PESTANAL™, analytical standard, ≥98.0%), poly(diallyldimethylammonium chloride) (PDADMAC, MW_av_: 100,000–200,000, 20 wt% in H_2_O, d = 1.040), and sulfuric acid (H_2_SO_4_, 95.0–97.0%) were purchased from Sigma-Aldrich (St. Louis, MO, USA). Titanium dioxide nanoparticles (nano-TiO_2_s, Aeroxide^®^ TiO_2_ P25, average primary particle size: ~21 nm, specific surface area: 50 ± 15 m^2^ g^−1^) were purchased from Evonik Industries AG (Essen, Germany). Stock Hg solution (1000 ± 6 μg mL^−1^, Hg metal in 2% HNO_3_) was purchased from High-Purity Standards (North Charleston, SC, USA). A certified reference material (CRM, Seronorm™ Trace Elements Urine L-2, freeze-dried human urine) was obtained from SERO (Billingstad, Norway).

### 2.2. Construction of the HPLC/Nano-TiO_2_-Coated Microfluidic-Based PCARD/ICP-MS System

**Figure 1:**
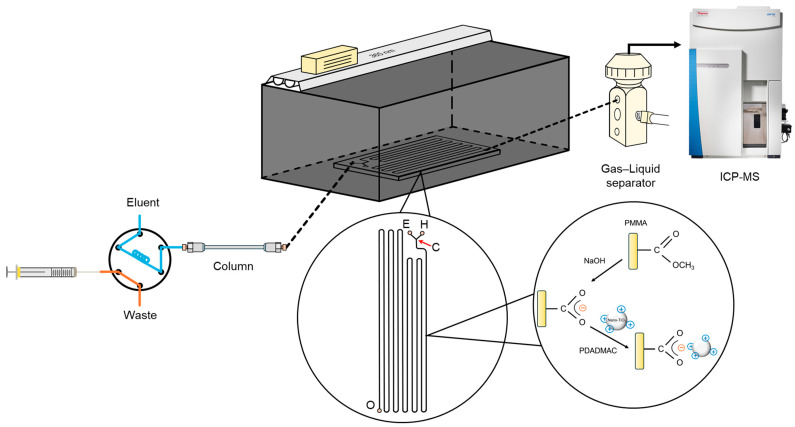
Schematic illustration of the HPLC/nano-TiO_2_-coated microfluidic-based PCARD/ICP-MS system.

A diagram of the HPLC/nano-TiO_2_-coated microfluidic-based PCARD/ICP-MS system is provided in Figure 1. The system can be divided into three main parts: the separation unit, the detection unit, and the VG unit interfaced with the two units described above.

The chromatographic separation unit consisted of an HPLC pump (S 1125-G, Sykam GmbH, Eresing, Germany), a six-port electric actuator valve (C22Z-3186E, VICI Valco Instruments Co. Inc., Houston, TX, USA) equipped with a 50-μL poly(aryletherketone) (PEEK) sample loop, and a guard column (XBridge^®^ BEH C18, 3.5 µm, 5 × 2.1 mm, Waters Corp., Milford, MA, USA) attached to an analytical column (XBridge^®^ C18, 3.5 µm, 150 × 3.0 mm i.d., Waters Corp., Milford, MA, USA). The detection was achieved by ICP-MS instrumentation (iCAP RQ, Thermo Fisher Scientific GmbH, Bremen, Germany).

The VG unit consisted of an in-house-fabricated nano-TiO_2_-coated microfluidic-based PCARD, an ultraviolet (UV) irradiation source (UV-A lamp, 40 W, maximum emission at 365 nm, Great Lighting Corp., New Taipei City, Taiwan) mounted in an opaque box, a mixing tee (Upchurch Scientific, Oak Harbor, WA, USA), and a gas–liquid separator (GLS) (B0507959, PerkinElmer Inc., Hopkinton, MA, USA). Briefly, the network of the microfluidic-based PCARD was designed using geometric modeling software (AutoCAD 2019, Autodesk Inc., Sausalito, CA, USA), then patterned on PMMA substrates (Kun Quan Engineering Plastics Co. Ltd., Hsinchu City, Taiwan) using a laser micromachining system (EBF-090060-60R, Laser Life Co. Ltd., Hsinchu City, Taiwan). Then, the channel interior of the developed device was modified with TiO_2_ photocatalysts via two-step dynamic coating procedures (using saturated NaOH for 12 h and a reagent containing 500 mg L^−1^ nano-TiO_2_ and 0.5% (*w*/*v*) PDADMAC for 8 h); Ultimately, the channel was flushed with high-purity water and dried under a gentle stream of air. Throughout the coating procedures, the operation flow rate was 0.1 mL min^−1^. Detailed descriptions of the fabrication procedures of the nano-TiO_2_-coated microfluidic-based PCARD are provided elsewhere [35].

All components of the units were connected by PEEK tubes (Upchurch Scientific Inc., Oak Harbor, WA, USA). Peristaltic pumps (Minipuls 3, Gilson Inc., Middleton, WI, USA) with peristaltic tubing (Gilson Inc., Middleton, WI, USA) were employed to deliver sample solutions and reagents. The outlet of the peristaltic tubing was modified for attachment to PEEK tubes via a conical adapter (Upchurch Scientific Inc., Oak Harbor, WA, USA).

### 2.3. Analytical Protocol

First, the sample was delivered into the chromatographic system for species separation. Afterward, the Hg species in the effluent were loaded into the nano-TiO_2_-coated microfluidic-based PCARD, followed by vaporization in the presence of HCOOH under UV irradiation (shown below) [23,36].

TiO_2_ + hν → TiO_2_ (holes (h^+^) + electrons (e^−^)) (1)

H_2_O ↔ H^+^ + OH^−^ (2)

OH^−^ + h^+^ → OH (3)

HCOOH + h^+^ → CO_2_ + H_2_O + mineral acids (4)

CH_3_Hg^+^_(aqueous)_ + OH‧ → Hg^2+^_(aqueous)_ + products (5)

Hg^2+^_(aqueous)_ + 2e^−^ → Hg^0^_(gas)_ (6)

Then the volatile Hg products were separated from the sample matrix via the GLS and carried into the ICP-MS system by a stream of Ar for subsequent measurement. (Caution! An exhaust system is recommended because of the generation of ozone and volatile Hg products during UV irradiation.) Adjustment of the sampling position and ion lenses for the optimal signal for Hg at *m*/*z* 202 was performed using an Hg standard solution. Detailed operational conditions for achieving optimal sensitivity and low background noise are provided in Table 1.

### 2.4. Characterization of the PDADMAC-Capped Nano-TiO_2_ Catalyst

The PDADMAC-capped nano-TiO_2_ catalyst was investigated using a particle analyzer (NanoBrook 90Plus PALS, Brookhaven Instrument Co., Holtsville, NY, USA) based on phase analysis light scattering (PALS). Test samples containing aliquots of nano-TiO_2_ and PDADMAC were dissolved in high-purity water and then adjusted to the desired pH using HNO_3_ and NaOH solutions. Data obtained during measurement were processed using built-in software (BIC Particle Solutions v. 3.6.0.7079 version 7.12.).

### 2.5. Sample Preparation

The urine samples were collected from volunteers in our research group. The water samples were collected from the water dispenser at the Department of Biomedical Engineering and Environmental Sciences, National Tsing Hua University (Hsinchu City, Taiwan) and effluents near industrial outfalls (New Taipei City, Taiwan). All samples were stored in glass bottles/vials (Yeong-Shin Co. Ltd., Hsinchu City, Taiwan) along with an aliquot of concentrated HCl, followed by wrapping with aluminum foil and storing at 4 °C in the dark [31]. The collected samples were filtered through a PTFE membrane (Acrodisc, 0.45 μm, 25 mm O.D., Pall Corp., Port Washington, NY, USA) before use. (Note: The bottles/vials were immersed in 40% HNO_3_ and then flushed with high-purity water. The bottles/vials were rinsed thoroughly with the designated samples prior to sample collection.)

## 3. Results and Discussion

### 3.1. Verification of the PDADMAC-Capped Nano-TiO_2_ Catalyst

**Figure 2:**
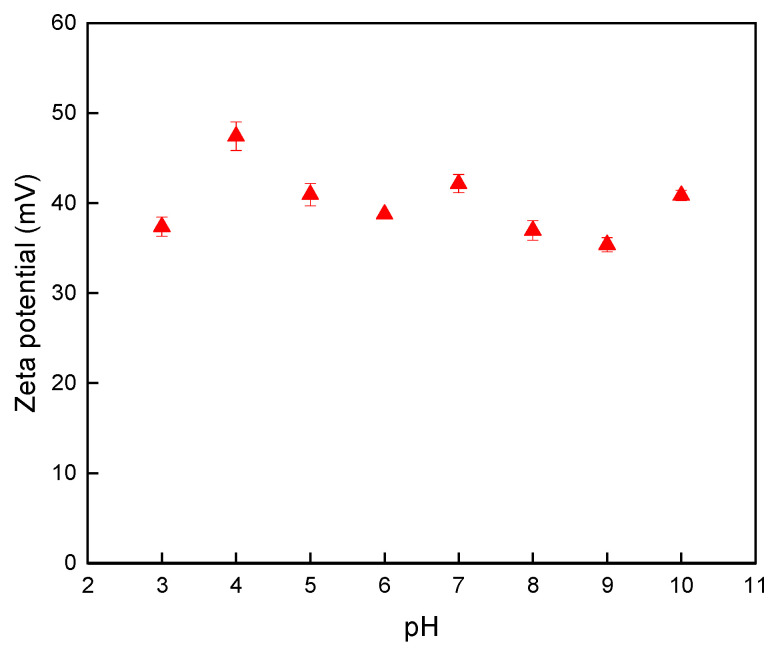
Variations in the zeta potentials of samples with respect to the pH of the solution. Uncertainty for each point shown by the error bar is expressed as standard deviation when *n* = 3.

Previously, numerous clusters of PDADMAC-capped nano-TiO_2_ catalysts forming a continuous bed in a channel interior have been validated by scanning electron microscope (SEM), energy dispersive X-ray analysis (EDAX), and a laser ablation (LA) system coupled with ICP-MS measurements [35]. Even so, the working theory of such a method was still not clearly elucidated. Thus, the causes of the unique characteristics of the PDADMAC-capped nano-TiO_2_ catalyst should be identified. Figure 2 presents the variations in the zeta potentials of the samples with respect to the pH of the solution. As indicated in Figure 2, no significant changes in the potentials of the samples were observed in the region ranging from 3.0 to 10.0, revealing that the strong cationic polyelectrolyte PDADMAC stabilized the nano-TiO_2_ catalyst against surrounding pH changes via cluster formation. As for the fluctuations in the zeta potentials in the investigated pH region, it might be attributed to variations in the ionic strengths of the tested solutions when the solutions were susceptible to significant dilution and pH adjustment [37,38]. Anyway, the PDADMAC-capped nano-TiO_2_s with a predominance of positive charges indeed provided a steady force for firm attachment to the negatively charged device channel treated, leading to high tolerance of flushing with strongly acidic and/or basic reagents.

In comparison with the TiO_2_ coating methods for PMMA substrates reported in the literature [39,40,41,42,43,44,45,46,47,48,49,50], both the preparation steps and additional equipment were dramatically simplified. Table 2 provides a comparison of the TiO_2_ coating methods for PMMA substrates proposed in this study with those reported in other studies. Furthermore, the amounts of chemicals used during the preparation procedures employed in this study were also much lower than those reported in other studies, which could be considered to fulfill the goals of green nanotechnology. According to our calculations, the consumption of the nano-TiO_2_ catalyst was approximately 24 mg. Remarkably, because the preparation conditions for the TiO_2_ coating were quite gentle, i.e., all processes are carried out in the aqueous phase and at room temperature, the morphology of the PMMA substrates was preserved. In other words, the methods employed in this study indeed provided a promising strategy for the development of nano-TiO_2_-enhanced, photoinduced VG in microfluidic devices due to the elimination of channel deformation.

### 3.2. Optimization of Operating Conditions for Chromatographic Separation

#### 3.2.1. Influence of L-Cysteine and 2-Mercaptoethanol Concentration on the Separation Efficiency of Hg Species

Because the nano-TiO_2_-coated microfluidic-based PCARD was used for vaporizing Hg species after the chromatographic separation, the operation conditions for chromatographic separation were an important issue for Hg speciation. Typically, the chromatographic separation of Hg species is achieved by reversed-phase methods and ion-exchange methods. Among the methods mentioned above, reversed-phase chromatography with stable analytical performance is especially popular [51]. In general, thiol-containing compounds (e.g., L-cysteine [52,53], 2-mercaptoethanol [54,55] or both [56]) are added into the mobile phase for complexing with Hg species (shown below).

R-SH + CH_3_Hg^+^ → R-S-Hg-CH_3_

R-SH + Hg^2+^ → R-S-Hg-S-R

Then, separation of each thiol-complexed Hg species can be achieved according to the mobility difference caused by varying degrees of interactions between thiol-complexed Hg species and the stationary phase.

**Figure 3:**
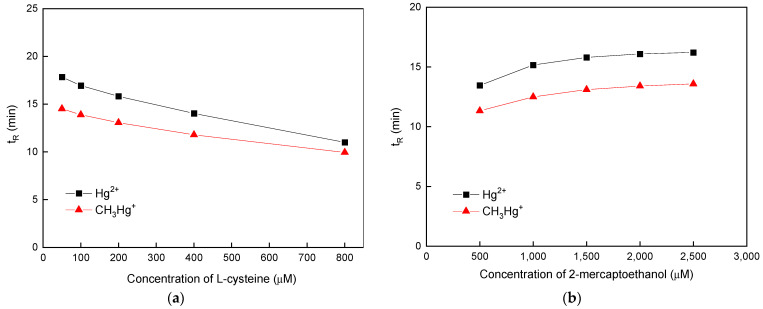
Variation in the retention time (t_R_) of the two Hg species with respect to the concentrations of (**a**) L-cysteine and (**b**) 2-mercaptoethanol. The uncertainty for each point shown by the error bar is expressed as standard deviation when *n* = 3.

Considering that the use of a single thiol-containing compound as a complexing reagent is often associated with a prolonged retention time (t_R_) [57], two thiol-containing compounds, i.e., L-cysteine and 2-mercaptoethanol, were simultaneously used for the separation of CH_3_Hg^+^ and Hg^2+^. Figure 3a,b displays the variation in t_R_ of the two Hg species as functions of L-cysteine and 2-mercaptoethanol concentration. As shown in Figure 3a,b, the maximum difference in t_R_ between CH_3_Hg^+^ and Hg^2+^ could be observed when 100 µM L-cysteine and 1500 µM 2-mercaptoethanol were applied. Therefore, the abovementioned concentrations of L-cysteine and 2-mercaptoethanol were selected for subsequent experiments.

#### 3.2.2. Influence of CH_3_OH Concentration on the Separation Efficiency of Hg Species

**Figure 4:**
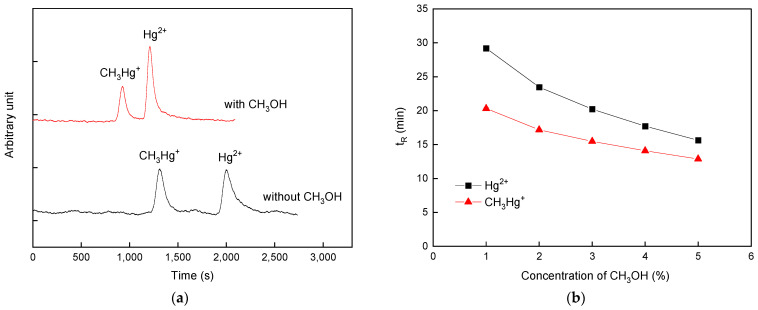
(**a**) Chromatograms of CH_3_Hg^+^ and Hg^2+^ under the conditions with/without CH_3_OH. (**b**) Variation in the retention time (t_R_) of the two Hg species with respect to the concentration of methanol. Uncertainty for each point shown by the error bar is expressed as standard deviation when *n* = 3.

Apart from the use of complexing reagents, the addition of CH_3_OH is thought to be another strategy for improving separation efficiency [58]. Figure 4a displays the chromatograms of CH_3_Hg^+^ and Hg^2+^ standards under conditions with the modifier CH_3_OH and without it. Compared to the chromatographic conditions without the addition of CH_3_OH, both the baseline stability and the signals profile were dramatically improved when CH_3_OH was applied. Furthermore, to ensure that the CH_3_OH added was favorable for both the analytical throughput and the separation efficiency, the influence of the CH_3_OH concentration on the retention behavior of the two Hg species was investigated. As shown in Figure 4b, a decreasing trend in both the t_R_ of the two Hg species and the difference in the t_R_ between CH_3_Hg^+^ and Hg^2+^ appeared when the CH_3_OH concentration was increased from 1 to 5%. Because undesired deposition resulting from excess organic modifiers in a sample matrix may cause permanent damage to ICP-MS instrumentation [59], a tradeoff between the analytical throughput and the separation efficiency was adopted by using a CH_3_OH concentration of 2% for the subsequent experiments. 

### 3.3. Optimization of Operating Conditions for Photocatalyst-Assisted VG

#### 3.3.1. Influence of HCOOH Concentration on the Vaporization Efficiency of Hg Species

**Figure 5:**
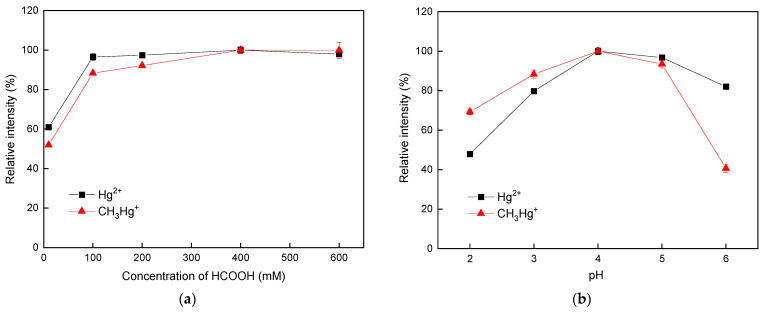
Variation in the signal intensity of the two Hg species with respect to (**a**) the concentration of HCOOH and (**b**) the pH. Uncertainty for each point shown by the error bar is expressed as standard deviation when *n* = 3. All the data were normalized to the maximal value.

Typically, photocatalytic reactions are triggered by electrons and holes when photocatalysts are treated with UV irradiation. The holes are generally considered to initiate oxidation reactions, whereas the electrons are responsible for photoreduction pathways. Because the photogenerated electrons and the holes may rapidly recombine, leading to inferior photocatalytic efficiency, additives that can retard the recombination of electrons and holes are usually used. In fact, low molecular weight organic substances such as HCOOH have been demonstrated to be useful for the improvement of the photocatalytic reduction efficiency of analytes due to their relatively high hole-scavenging efficiency [60]. To determine the optimal concentration of HCOOH for the reduction of Hg species, the influence of HCOOH concentration on the signal intensity of the analytes was evaluated. As shown in Figure 5a, significant enhancements in the signals of the two Hg species were obtained when HCOOH was added, reaching a plateau at the HCOOH concentration of 400 mM. Therefore, an optimal HCOOH concentration of 400 mM, which provided maximum signals for CH_3_Hg^+^ and Hg^2+^, was selected for subsequent experiments.

#### 3.3.2. Influence of the pH on the Vaporization Efficiency of Hg Species

It has been recognized that adsorption among analytes of interest, hole scavengers (i.e., HCOOH), and nano-TiO_2_ catalysts via electrostatic interactions is the key to success in the photocatalyst-assisted reduction reaction. Adsorption among target analytes, hole scavengers, and nano-TiO_2_ catalysts via electrostatic interactions is considered critical to the efficiency of the photocatalyst-assisted reduction reaction. Because the charge statuses of all species were strongly determined by the acidity of the reaction environment, the pH of the mixture resulting from the column effluent and the hole scavenger was investigated. As indicated in Figure 5b, an increasing trend in the intensity of the two Hg species appeared when the pH was increased from 2.0 to 4.0. In contrast, a deterioration in the signal intensity for both Hg species was observed as the pH exceeded 5.0. This phenomenon could be explained in terms of the degree of deprotonation of HCOOH, leading to competitive adsorption between the analyte species and HCOOH on nano-TiO_2_ photocatalysts. The phenomenon might also be attributed to competitive adsorption between the analyte species and HCOOH on nano-TiO_2_ photocatalysts caused by varying degrees of deprotonation of HCOOH. Therefore, the optimal value of pH 4.0, which provided the highest intensity signals for CH_3_Hg^+^ and Hg^2+^, was selected for subsequent experiments.

### 3.4. Analytical Performance

**Figure 6:**
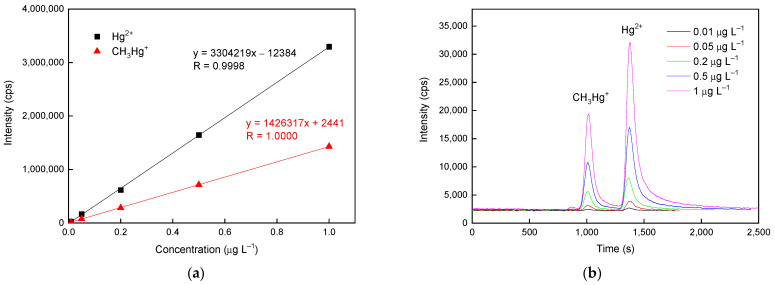
(**a**) Calibration curves and (**b**) chromatograms of the two Hg species obtained using the HPLC/nano-TiO_2_-coated microfluidic-based PCARD/ICP-MS system.

Table 3 presents the analytical features of merit of the established system operated under optimum conditions. Satisfactory linearities in the region from 0.01 to 1.0 μg L^−1^ for the two Hg species were observed, with correlation coefficients higher than 0.9998 (see Figure 6a). Figure 6b displays the corresponding chromatograms under the optimized conditions. The method detection limits (MDLs) for CH_3_Hg^+^ and Hg^2+^ were 2.95 and 1.39 ng L^−1^, respectively. (Note: The MDLs were determined based on the 3σ criterion, where the standard deviation was obtained from the results of seven repeated measurements of a mixture of column effluent.) Moreover, 15 replicate injections of 0.2 μg L^−1^ samples of each species were conducted to estimate both the stability of the system during the analytical procedures and the drift of the Hg response caused by the memory effect. The obtained repeatability was less than 3% of the coefficient of variation (CV), demonstrating the precision of this method for durable analyses. Then, the accuracy of the proposed method was validated by analysis with the CRM Seronorm™ Trace Elements Urine L-2. Because detailed concentration information regarding Hg species in the CRM was absent, comparison between the certified value of Hg concentration and the summation of the measured values of individual species was adopted. Based on the analytical results, the summation of each measured value was in reasonably good agreement with the certified Hg concentration (see Table 3). Moreover, the CRM with intentional 1000-fold dilution was employed to verify the detection capability of the established system for quantitatively determining ultratrace levels of Hg species.

To further demonstrate the utility of the proposed system, urine samples obtained from three healthy volunteers and water samples collected from the water dispenser and effluents near industrial outfalls were analyzed under the optimized operation conditions. As indicated in Table 4, the concentrations of Hg^2+^ ranged from 0.036 to 0.112 μg L^−1^ in the collected samples, and the CH_3_Hg^+^ content was much lower than the Hg^2+^ content in either urine samples or water samples. It is hypothesized that such diverse distributions of CH_3_Hg^+^ and Hg^2+^ are closely related to the unique metabolic mechanisms of the two species [1,7]. Even so, the concentration information for the two Hg species, i.e., CH_3_Hg^+^ and Hg^2+^, could be simultaneously obtained by the proposed method. The acceptable spike recovery of Hg species also revealed that the method employed in this study would be feasible for Hg speciation.

## 4. Conclusions

In this study, a selective and sensitive hyphenated system including the nano-TiO_2_-coated microfluidic-based PCARD, HPLC and ICP-MS for Hg speciation was established. To fabricate the nano-TiO_2_-coated microfluidic-based PCARD, an alternative involving a charge-rich polyelectrolyte PDADMAC was employed to stabilize the nano-TiO_2_ catalyst via cluster formation and provide a steady force for firm attachment to the channel interior via electrostatic interactions. The MDLs for CH_3_Hg^+^ and Hg^2+^ achieved by the established system were 2.95 and 1.39 ng L^−1^, respectively, demonstrating the capability of such a system for quantitatively determining ultratrace levels of Hg species. A series of validation experiments in terms of precision, accuracy, and so on, indicated that the method could also be satisfactorily applied to the determination of the two Hg species in both human urine and water samples. Remarkably, the durability of the nano-TiO_2_-coated microfluidic-based PCARD created by the unique properties of the PDADMAC-capped nano-TiO_2_ catalyst was also verified.

## Figures and Tables

**Table 1:** Operational conditions for the HPLC/nano-TiO_2_-coated microfluidic-based PCARD /ICP-MS system.

Chromatographic Separation	
chromatographic column	XBridge^®^ C18, 3.5 µm, 150 × 3.0 mm i.d.
mobile phase solution	2% CH_3_OH, 100 μM L-cysteine, 1500 μM 2-mercaptoethanol, 10 mM CH_3_COONH_4_, pH 4
separation flow rate	0.3 mL min^−1^
sample volume	50 μL
Nano-TiO_2_-Coated Microfluidic-Based PCARD	
dimension of reaction channel	544 mm (W) × 907 mm (D) × 26mm (L)
hole-scavenger reagent resulting mixture for photoreduction	400 mM HCOOH, pH 4, 1 mL min^−1^
reaction time	15 s
illumination density	10 mW cm^−2^
iCAP RQ ICP-MS Detection	
plasma power	1550 W
cool flow	14 L min^−1^ Ar
auxiliary flow	0.8 L min^−1^ Ar
nebulizer gas	1.065 L min^−1^ Ar
sampling cone	nickel
skimmer cone	nickel

**Table 2:** Comparison of the TiO_2_ coating method for PMMA substrates proposed in this study with those previously reported in the literature.

Coating Mechanism	Chemicals	Substrate Form	Incubation Temperature	Step	Additional Equipment	Citation
electrostatic attraction	NaOH, TiO_2_^a^, PDADMAC, high-purity water	channel	R.T. ^b^	2	peristatic pump	this study
covalent bonding	hexamethylene diamine, borate buffer, glutaraldehyde, phosphate buffer, dopamine hydrochloride, dimethyl formamide, TSU ^c^, DIPA ^d^, TiO_2_^a^, glycidyl isopropyl ether, NaCl, tris-EDTA buffer, DNA	sheet	R.T.–94 °C	9		[39]
sol-gel entrapment	TiCl_4_^a^, tert-butanol	powder	R.T.–75 °C	4	rotary evaporator, oven	[40]
sol-gel entrapment	AIBN ^e^, TiO_2_^a^	monomer	40–50 °C	3	oven, centrifuge	[41]
sol-gel entrapment	ethanol, CH_2_Cl_2_, Ti(C_4_H_9_)_4_^a^, glacial acetic acid	powder	R.T.–135 °C	6	Teflon-lined stainless-steel, oven, electrospinning system	[42]
sol-gel entrapment	TiO_2_^a^, methacrylic acid, isopropanol	powder	80–85 °C	5	stereolithography (SLA) 3D printer	[43,44]
sol-gel entrapment	TiO_2_^a^, acetone, ethyl lactate, ethanol, diazonaphtoquinone	powder	80 °C	2	spin coater/screen-printer, oven	[45]
sol-gel entrapment	TiO_2_^a^, triethyl phosphate	powder	R.T.	3	manual casting knife	[46]
sol-gel entrapment	N-TiO_2_^f^, iso-butanol	sheet	80 °C	3	dip coater, ultrasonicator	[47]
adhesive	Ti[OCH(CH_3_)_2_]_4_^a^, colloidal SiO_2_, HClO_4_, absolute ethanol, tetraethyl orthosilicate, HCl, isopropanol, propanol, 2-propoxyethanol	sheet	R.T.	4	Heat gun, dip coater	[48]
adhesive	TiO_2_^a^, Ti_4_O_7_^a^, acetone, silicon-based commercial glue	sheet	30 °C	3	oven	[49]
deposition	Ti[OCH(CH_3_)_2_]_4_^a^	sheet	25–50 °C	1	atmospheric pressure plasma jet generator	[50]

^a^ Commercially available products. ^b^ Room temperature. ^c^ O-(N-Succinimidyl)-N,N,N,N-tetramethylammonium tetrafluoroborate. ^d^ N,N-diisopropyl amine. ^e^ Azobisisobutyronitrile. ^f^ In-lab-prepared products.

**Table 3:** Analytical characteristics of the proposed HPLC/nano-TiO_2_-coated microfluidic-based PCARD /ICP-MS system.

Species	Linear Equation	R^2 a^	Linear Range, μg L^−1^	MDL ^b^, ng L^−1^	Precision ^c^, %	Seronorm Trace Elements Urine L-2 (Freeze-Dried Human Urine)		
						Certified Value, μg L^−1^	Measured Value ^d^, μg L^−1^	Spike Recovery, %
CH_3_Hg^+^	y = 1426317x + 2441	1.0000	0.01–1	2.95	1	39.8 ± 8.0	N.D. ^e^	107 ^f^
Hg^2+^	y = 3304219x − 12384	0.9998	0.01–1	1.39	3		41.4 ± 0.4	106 ^f^

^a^ Correlation coefficient. ^b^ Method detection limit; sample volume = 50 μL; *n* = 7. ^c^ Relative standard deviation; standard concentration: 0.2 μg L^−1^ (*n* = 15). ^d^ Mean ± standard deviation (*n* = 3). ^e^ Not detected. ^f^ Spiked concentration = 0.1 μg L^−1^.

**Table 4:** Analysis of urine and water samples.

Sample	CH_3_Hg^+^		Hg^2+^	
	Measured Value ^a^, μg L^−1^	Spike Recovery, %	Measured Value, μg L^−1^	Spike Recovery, %
Urine 1	N.D. ^b^ (N.D.) ^c^	99 ^d^	0.112 ± 0.004 (1.12 ± 0.04)	95 ^d^
Urine 2	N.D. (N.D.)	108 ^e^	0.057 ± 0.002 (0.57 ± 0.02)	113 ^e^
Urine 3	N.D. (N.D.)	94 ^d^	N.D. (N.D.)	102 ^d^
Drinking water	N.D. (N.D.)	92 ^f^	N.D. (N.D.)	96 ^f^
Effluent water 1	N.D. (N.D.)	106 ^f^	N.D. (N.D.)	97 ^f^
Effluent water 2	N.D. (N.D.)	114 ^g^	0.036 ± 0.002 (0.072 ± 0.004)	116 ^g^

^a^ Mean ±standard deviation (*n* = 3). ^b^ Not detected. ^c^ Values in parentheses are the concentration of species in original samples. ^d^ Spiked concentration = 0.1 μg L^−1^. ^e^ Spiked concentration = 0.05 μg L^−1^. ^f^ Spiked concentration = 0.02 μg L^−1^. ^g^ Spiked concentration = 0.04 μg L^−1^.

## Data Availability

All data generated or analyzed during this study are included in this manuscript.
